# EUS-guided pancreaticoduodenostomy using a lumen-apposing metal stent as a primary approach to treat difficult pancreatolithiasis: creating a side door to unlock the front door

**DOI:** 10.1016/j.vgie.2024.09.009

**Published:** 2024-09-11

**Authors:** Michael Lajin, Cainan Foltz, Hong-Der Lin, Michael Romero, Kian Bagheri

**Affiliations:** Sharp HealthCare, Division of Gastroenterology and Hepatology, Department of Internal Medicine, San Diego, California, USA

## Introduction

Pancreatic duct (PD) hypertension resulting from obstructive stones/strictures can cause abdominal pain that may respond to decompressing the PD in selected patients with chronic pancreatitis.^1^^,^^2^ Challenges to treating pancreatolithiasis include large, calcified, and compacted stones inside PD strictures.^2^^,^^3^

Treatment options in such scenarios are surgical, endoscopic, and extracorporeal shockwave lithotripsy.^4^ EUS-guided biliary drainage and pancreatic drainage (EUS-PD) have emerged as rescue treatments for difficult biliary^5^ and pancreatic^2^^,^^3^^,^^6^ stones. Although a first-intent EUS-guided biliary drainage was described in malignant biliary obstructions,^7^ there are no similar reports of primary EUS-PD.

EUS-PD is typically a wire-guided creation/dilation of a tract followed by deploying transgastric or transduodenal plastic stents.^6^ This technique carries inherent risks such as leaks, bleeding, stent dysfunction, or migration.^6^^,^^8^ We describe a first-intent EUS-PD using a lumen-apposing metal stent (LAMS) deployed with a freehand technique to treat difficult pancreatolithiasis.

## Case report

**Figure 1:**
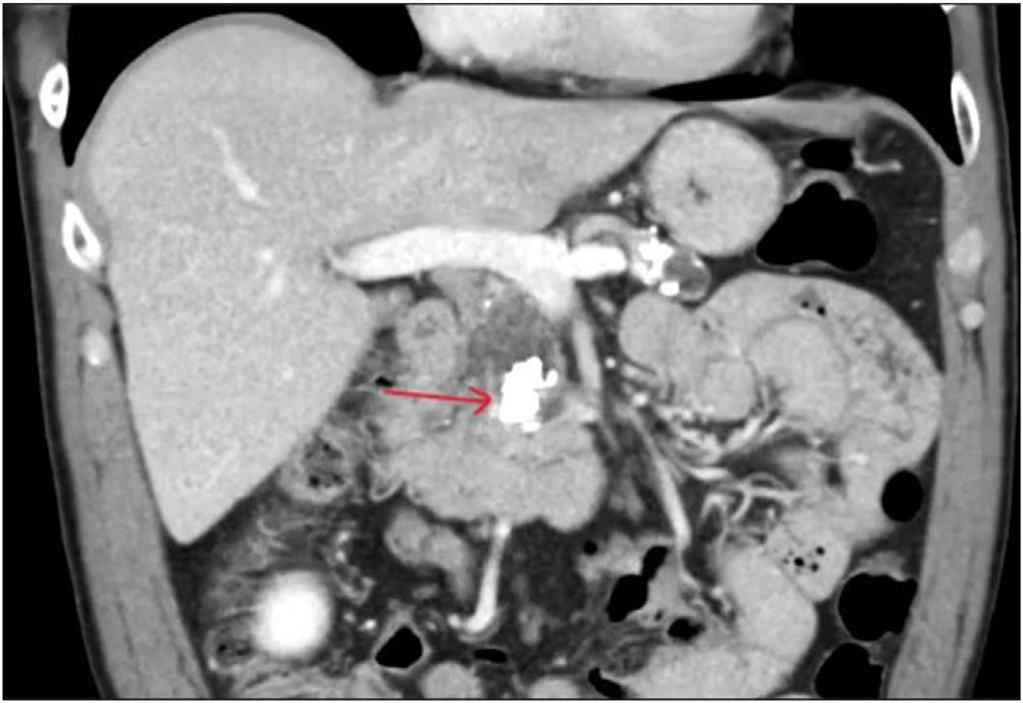
CT image demonstrating a large burden of calcified obstructive pancreatic stones impacted above the papilla (*red arrow*).

A 55-year-old man with non–insulin-dependent diabetes and alcoholic chronic calcific pancreatitis presented with abdominal pain and a 12-pound weight loss. CT of the abdomen and MRCP showed a large burden of calcified obstructive PD stones above the papilla (Fig. 1) resulting in upstream PD dilatation measuring 19 mm.

We discussed the options including surgical, endoscopic, and referral for extracorporeal shockwave lithotripsy (unavailable in our institution). He elected to proceed with endoscopic treatment.

**Figure 2:**
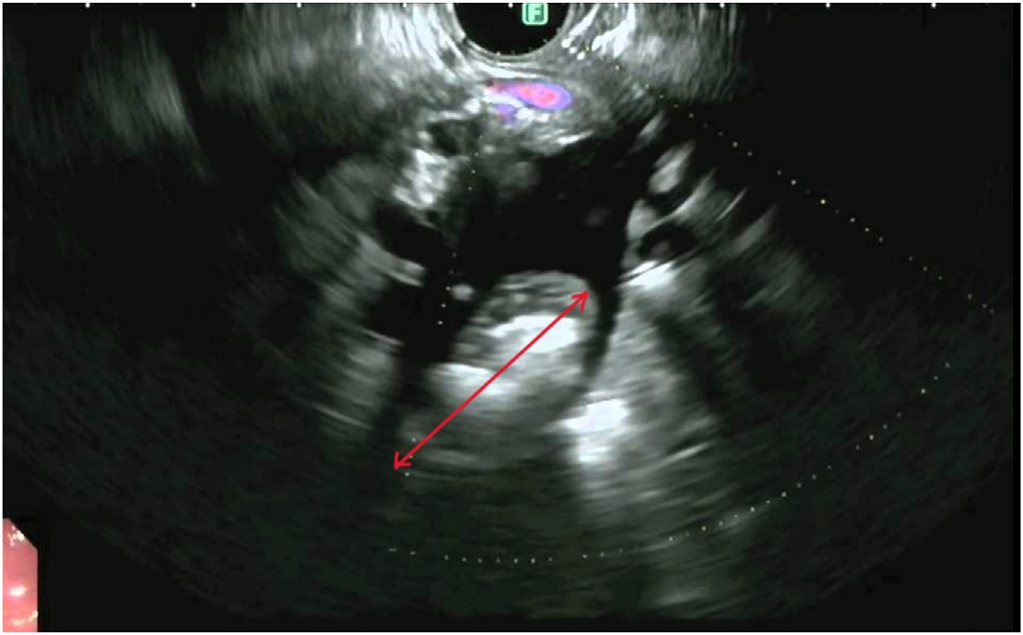
EUS image demonstrating a dilatated pancreatic duct and a large conglomerate of obstructive stones (*red double arrow*) at the head of the pancreas.

EUS demonstrated a dilated PD and a large conglomerate of obstructive stones at the head of the pancreas (Fig. 2). Because of the size of stones compacted above the papilla, we decided to perform primary EUS-PD, given the high chance of a failed ERCP in this setting. This decision was made after a multidisciplinary discussion with the surgical team.

An electrocautery-enhanced LAMS was used because of sufficient dilatation of the PD allowing a safe deployment and the minuscule distance separating the PD from the GI wall.

LAMSs possess favorable characteristics for EUS-PD. Its electrocautery-enhanced delivery system allows the stent catheter to penetrate a fibrous PD wall. It is fully covered with an antimigration structure. The stent deployment mechanism avoids antecedent tract creation/dilation. In addition, LAMSs have a wider lumen. These features might minimize adverse events linked to the procedure.

**Figure 3:**
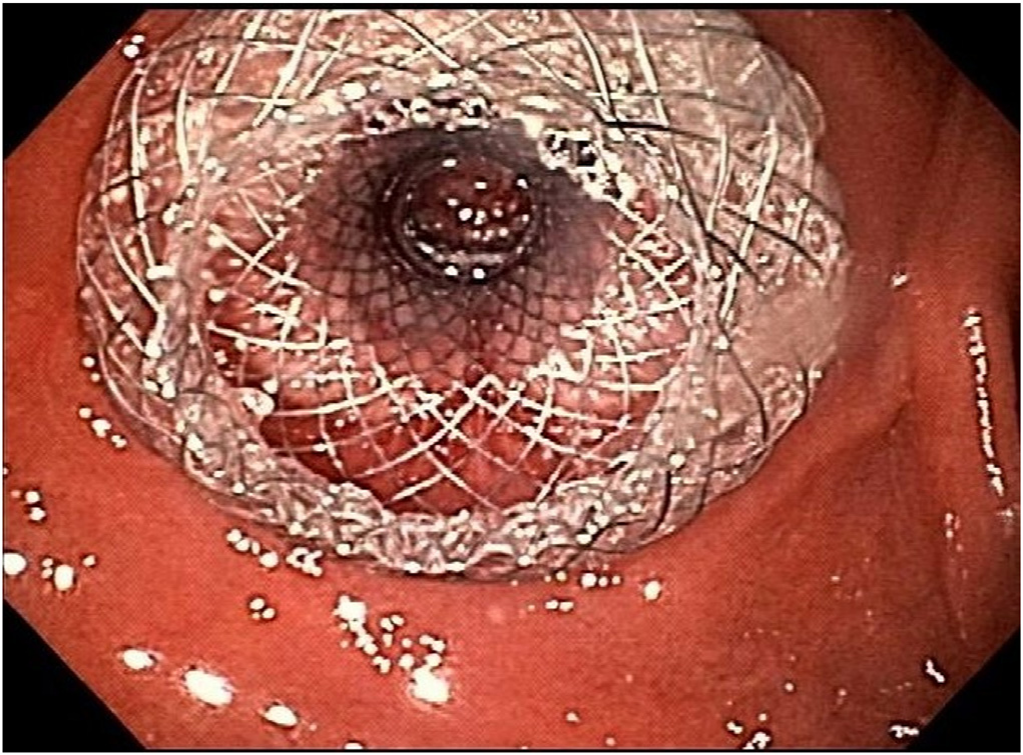
Endoscopic image of a lumen-apposing metal stent connecting the duodenal bulb to the pancreatic duct.

**Figure 4:**
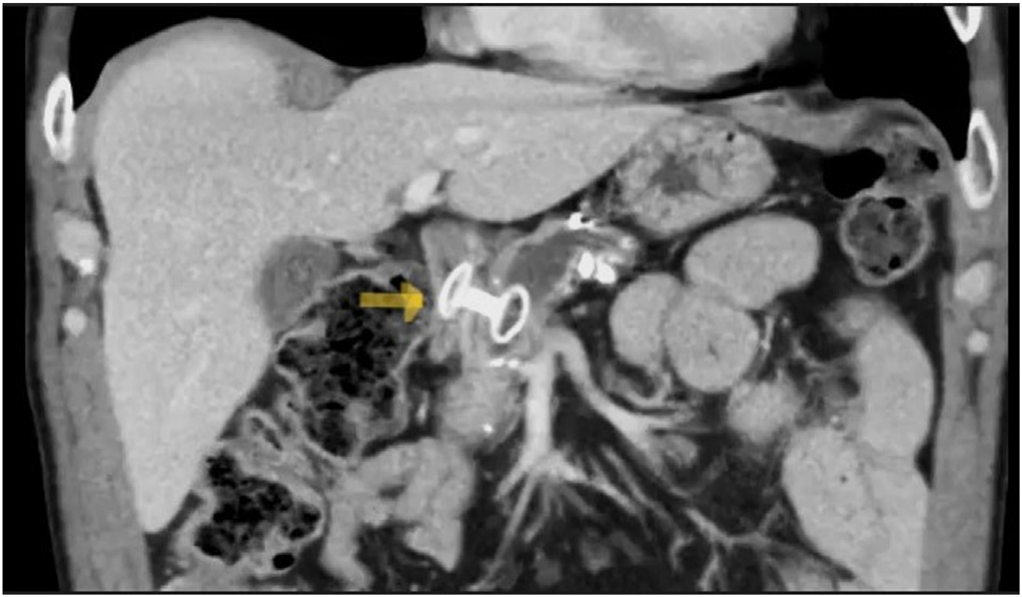
CT image of a lumen-apposing metal stent at the pancreaticoduodenostomy (*yellow arrow*).

The duodenal bulb provided more stability for the echoendoscope with no intervening vessels. Using electrocautery, we deployed the LAMS (10 × 10 mm) with a freehand technique achieving a pancreaticoduodenostomy (Fig. 3). Decompression of the PD was achieved as shown on CT (Fig. 4), resulting in substantial pain relief for the patient.

**Figure 5:**
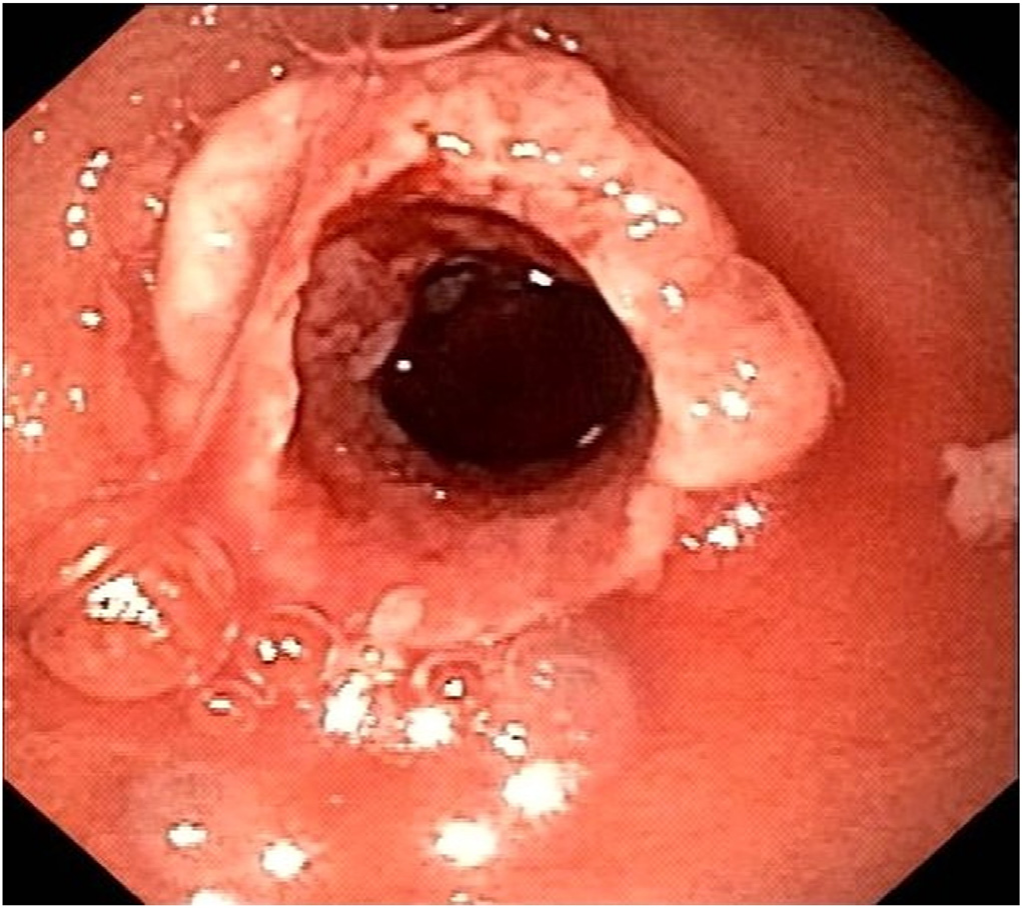
Endoscopic image of the pancreaticoduodenal fistula.

**Figure 6:**
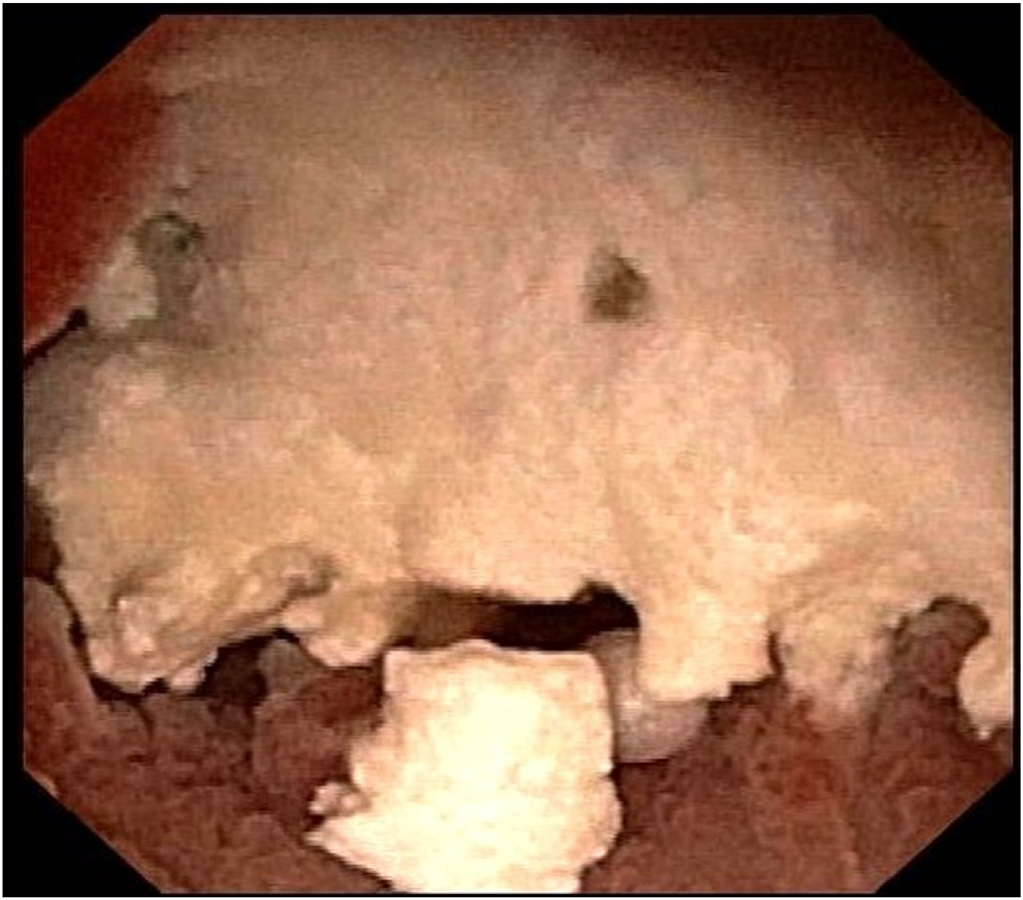
Large, calcified, and compacted pancreatic duct stones visualized by the pediatric endoscope.

**Figure 7:**
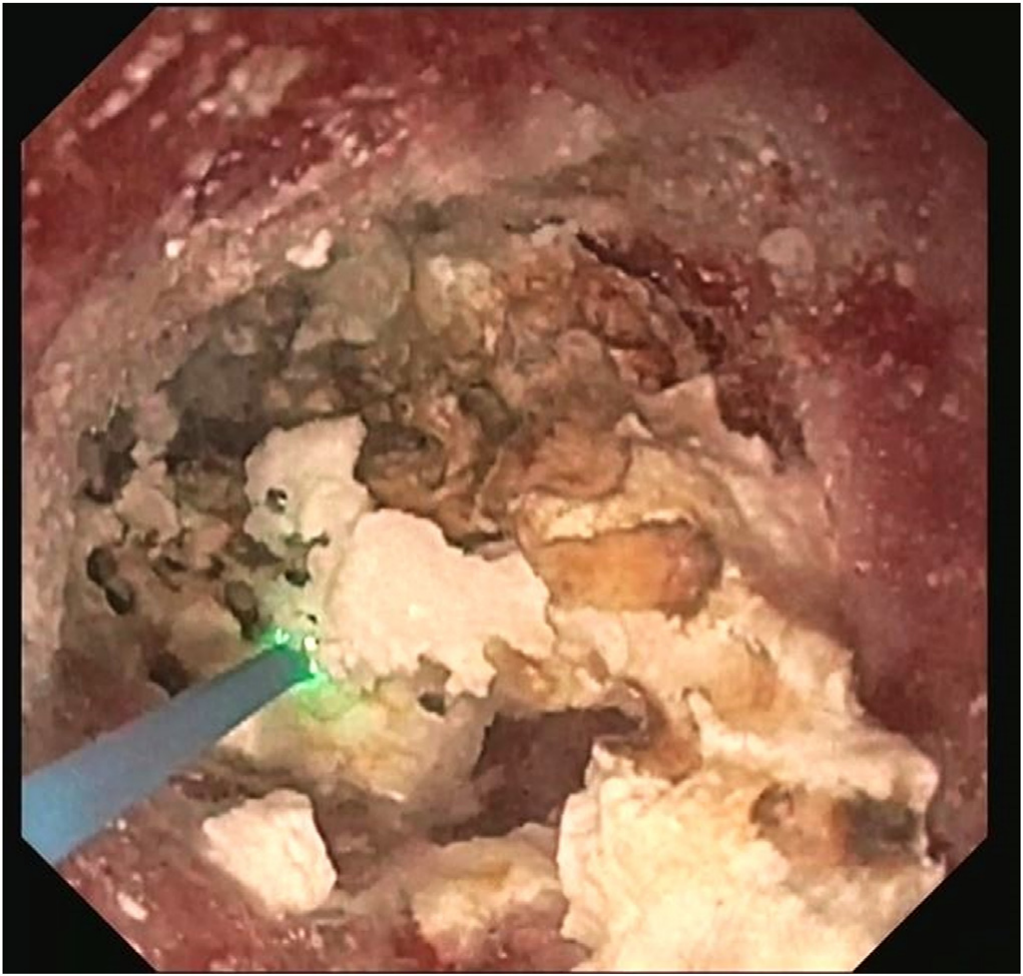
Laser lithotripsy under direct visualization using a pediatric endoscope through the fistula.

Six weeks later, the LAMS was removed. A pediatric endoscope (GIF-XP190N/5-mm outer diameter; Olympus, Tokyo, Japan) was advanced through the fistula (Fig. 5) inside the PD toward the head of the pancreas. Saline immersion was used to avoid the risk of CO_2_ embolization. Large impacted stones (Fig. 6) were fragmented with laser lithotripsy (15 W, 12 Hz) (Fig. 7). Laser lithotripsy compared with electrohydraulic lithotripsy has been shown to have a greater technical and clinical success.^9^ After extensive lithotripsy, a new LAMS was placed to maintain access.

Four weeks later, the LAMS was removed. Stone fragments were extracted using forceps until a stricture was encountered. The stricture prevented further advancement of the pediatric endoscope.

**Figure 8:**
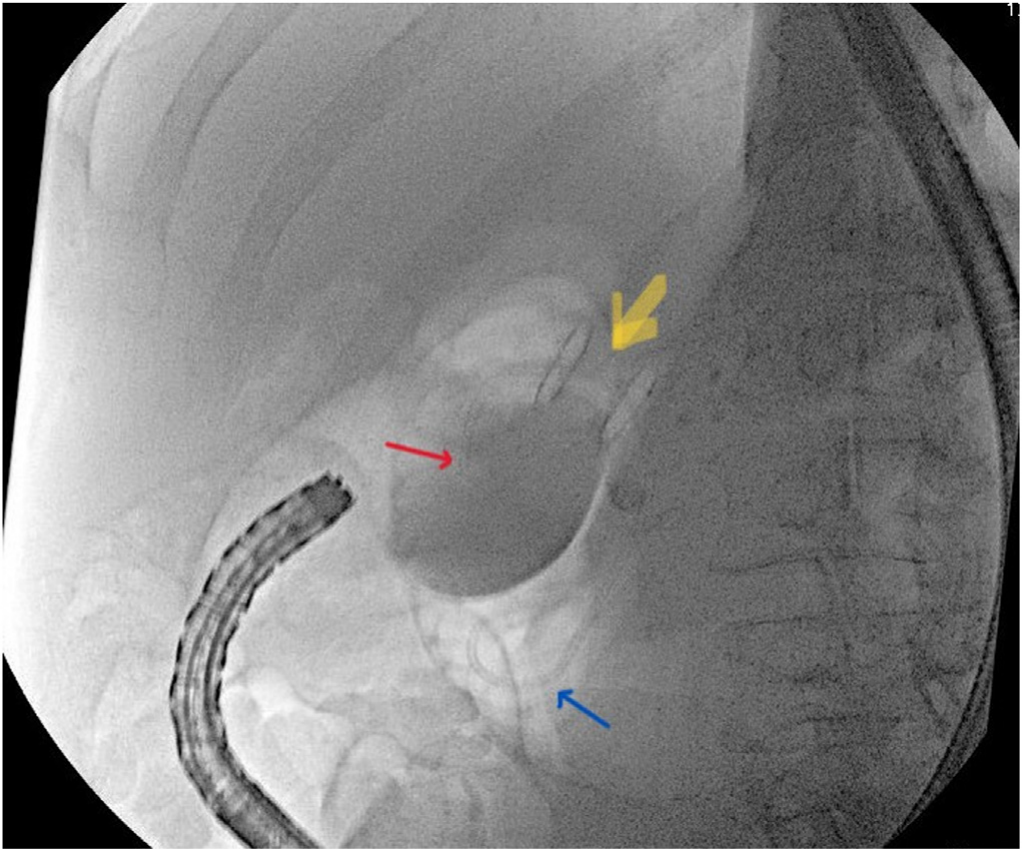
Lumen-apposing metal stent deployed across the fistula (*yellow arrow*) and through it, a double-pigtail stent deployed with one end in the duodenal bulb (*red arrow*) and the other end exiting the papilla to the descending duodenum (*blue arrow*).

After we switched to a therapeutic endoscope, an ERCP catheter with a guidewire was advanced through the fistula and maneuvered downstream exiting the papilla. The strictures were dilated with a 6-mm balloon. The LAMS was deployed across the fistula. Through the LAMS, we deployed a double-pigtail stent (7F, 7 cm) entering the fistula and exiting the papilla (Fig. 8) to dilate the stricture.

Three months later, ERCP was performed. The PD was cannulated with a wire entering the papilla and exiting the fistula. The stent was removed followed by pancreatic sphincterotomy and balloon dilation.

Pancreatoscopy revealed large stones. The stones were fragmented with laser lithotripsy. Fragments were flushed or extracted until the duct at the head of the pancreas was cleared. Because of this, there was no further need to replace the LAMS that was removed during this procedure. The wire was redirected toward the tail of the PD. A flanged stent (7F, 12 cm) was deployed.

Four weeks later, the pancreatic stent was cannulated with a wire. A slightly opened snare was threaded over the wire, grasping and removing the pancreatic stent as the wire was kept in place. A pancreatogram showed persistent downstream stricture and filling defects at the tail.

**Figure 9:**
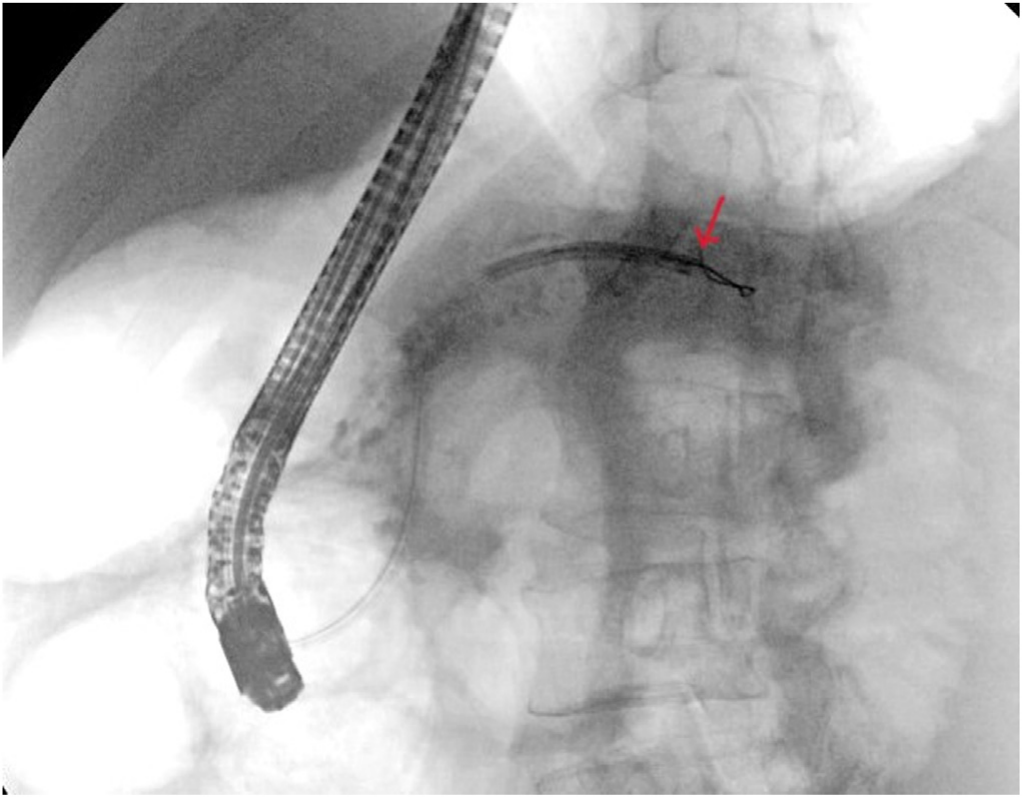
Fluoroscopic image of the pancreatoscope advanced through the papilla toward the tail of the pancreatic duct (*red arrow*).

After balloon dilation, pancreatoscopy at the tail (Fig. 9) revealed stones. These stones were fragmented with laser lithotripsy and extracted with a balloon. Clearance of the entire PD was confirmed on pancreatoscopy. A stent (10F, 13-cm) was deployed.

## Outcome

The patient had no adverse events aside from flares of abdominal pain after lithotripsy sessions requiring short hospitalizations (23-48 hours). This occurred despite the administration of prophylactic antibiotics, rectal indomethacin, and intravenous hydration. Otherwise, his abdominal pain and quality of life improved substantially. He tolerates a low-fat diet and is maintaining his weight.

## Conclusions

Although this case report demonstrates the feasibility of performing EUS-guided pancreatic drainage as a primary approach to treating difficult pancreatolithiasis in experienced centers, more data are needed before recommending the adoption of this approach. Using a LAMS to perform EUS-guided PD drainage might offer several advantages over the traditional tract creation/dilation technique, such as less risk of migration and leak. Nevertheless, this approach is only feasible when the PD dilatation is sufficient to allow safe deployment of the stent (Video 1, available online at www.videogie.org).

## Patient consent

The patient in this article has given written informed consent to publication of the case details.

## Disclosure

Michael Lajin disclosed paid sub-investigator for Fractyl Health. All other authors disclosed no financial relationships.

## Supplementary data

Video 1 EUS-guided pancreaticoduodenostomy using lumen-apposing metal stent as a primary approach to treat difficult pancreatolithiasis.

## References

[1] Poulsen J.L., Olesen S.S., Malver L.P. (2013). Pain and chronic pancreatitis: a complex interplay of multiple mechanisms. World J Gastroenterol.

[2] Chapman C.G., Waxman I., Siddiqui U.D. (2016). Endoscopic ultrasound (EUS)-guided pancreatic duct drainage: the basics of when and how to perform EUS-guided pancreatic duct interventions. Clin Endosc.

[3] Baars J.E., Chen F., Sandroussi C. (2018). EUS-guided pancreatic duct drainage: approach to a challenging procedure. Endosc Ultrasound.

[4] Kim Y.H., Jang S.I., Rhee K. (2014). Endoscopic treatment of pancreatic calculi. Clin Endosc.

[5] Lajin M. (2022). EUS-guided hepaticogastrostomy using a rendezvous technique to treat left intrahepatic duct stones in a patient with recurrent pyogenic cholangitis. VideoGIE.

[6] Tessier G., Bories E., Arvanitakis M. (2007). EUS-guided pancreatogastrostomy and pancreatobulbostomy for the treatment of pain in patients with pancreatic ductal dilatation inaccessible for transpapillary endoscopic therapy. Gastrointest Endosc.

[7] Chen Y.I., Sahai A., Donatelli G. (2023). Endoscopic ultrasound-guided biliary drainage of first intent with a lumen-apposing metal stent vs endoscopic retrograde cholangiopancreatography in malignant distal biliary obstruction: a multicenter randomized controlled study (ELEMENT Trial). Gastroenterology.

[8] Imoto A., Ogura T., Higuchi K. (2020). Endoscopic ultrasound-guided pancreatic duct drainage: techniques and literature review of transmural stenting. Clin Endosc.

[9] Saghir S.M., Mashiana H.S., Mohan B.P. (2020). Efficacy of pancreatoscopy for pancreatic duct stones: a systematic review and meta-analysis. World J Gastroenterol.

